# Prevalence of antibodies against *Leptospira* sp. in snakes, lizards and turtles in Slovenia

**DOI:** 10.1186/1751-0147-55-65

**Published:** 2013-09-10

**Authors:** Renata Lindtner-Knific, Aleksandra Vergles-Rataj, Ksenija Vlahović, Petra Zrimšek, Alenka Dovč

**Affiliations:** 1 Veterinary Faculty, Institute for Health Care of Poultry, University of Ljubljana, Gerbičeva 60, 1000, Ljubljana, Slovenia; 2 Veterinary Faculty, Institute for Microbiology and Parasitology, University of Ljubljana, Gerbičeva 60, 1000, Ljubljana, Slovenia; 3 Veterinary Faculty, Department of Biology, University of Zagreb, Heinzelova 55, 10000, Zagreb, Croatia; 4 Veterinary Faculty, Clinic for Reproduction and Horses, University of Ljubljana, Gerbičeva 60, 1000, Ljubljana, Slovenia

**Keywords:** Serology, Antibody, Leptospira, Diagnosis, Reptiles

## Abstract

**Background:**

Leptospiral infections in poikilothermic (cold blooded) animals have received very little attention and the literature concerning natural infections of these animals is limited. The aim of this study was to determine the prevalence of leptospiral antibodies in reptiles, imported into Slovenia and intended to be pets in close contact with humans. A total of 297 reptiles (22 snakes, 210 lizards and 65 turtles) were tested for specific antibodies against serovars of *Leptospira interrogans* sensu stricto using the microscopic agglutination test (MAT). Live cultures of different serovars were used as antigens. MAT was performed according to standard procedures and the degree of reaction was interpreted by estimating the percentage of agglutinated leptospires. Samples showing titres of ≥ 50 against one or more serovars were considered as positive.

**Results:**

Antibodies against seven pathogenic serovars of *L. interrogans* sensu stricto were detected in 46 of 297 reptiles. Among 22 snakes, specific antibodies against pathogenic serovars of three *Leptospira* species (*L. interrogans*, *L. kirschneri* and *L. borgpetersenii*) at titre levels from 1:50 to 1:400 were detected in 6 snakes. In 31 of 210 lizards, specific antibodies were found in titres from 1:50 to 1:1000 and, finally, among 65 turtles (terrapins and tortoises), 9 had specific antibodies at titre levels between 1:50 and 1:1600. Animals imported from non-EU countries showed significantly higher prevalence (25.0%; 95 confidence interval: 16.7–33.3%) than animals from EU member states (10.4%; confidence interval: 6.1–14.7%).

**Conclusions:**

Reptiles may be considered as potential reservoirs of *L. interrogans* sensu stricto. Origin of the animals is a risk factor for presence of leptospiral antibodies, especially in lizards. Special attention should be focused on animals from non-EU member states.

## Background

Leptospiral infections in poikilothermic (cold blooded) animals have received very little attention and the literature concerning natural infections of these animals is limited. Authors have reported leptospiral antibodies in reptiles, mostly in animals collected during studies focusing on warm blooded animals [1-4]. Isolation of leptospires has generally been unsuccessful due to the contamination of inoculated materials [5], but Ferris *et al.*[6] succeeded to isolate leptospires from Hog-nosed snakes (*Heterodon platyrhinos*).

Since the first diagnosed leptospiral infections in humans, many researchers have tested various species of wild and domestic animals for the bacteria or antibodies. By the use of serological tests, specific antibodies against pathogenic serovars similar to those found in animals and humans have been found in reptiles from the same environment. The role of cold blooded animals in the maintenance and continuity of leptospires in the environment and as a source of spreading the infection to warm blooded species is still poorly understood, but the relationship between *Leptospira* spp.*,* reptiles and amphibians may be important for the epidemiology of leptospirosis. Leptospirosis is a waterborne infection. Andrews *et al.*[7], found high antibody titres in Terrapins (*Trachemys scripta elegans*) and postulated that high antibody titres develop under natural conditions as the result of long term exposure to leptospires in water. It has not yet been established whether pathogenic eptospires remain pathogenic after being excreted into water or whether they become saprophytic or eventually disintegrate and die [8].

The origin of pet reptiles in trade is often unknown; they may be bred in captivity, born by wild-caught parents or taken directly from nature. Reptiles have become common domestic pets and may carry zoonotic infections such as salmonellosis [9]. The results of various studies on leptospiral infection of poikilothermic animals conducted by several authors suggest that leptospirosis in these animals are not negligible [5]. Based on literature, the results of our previous study [10] and the fact that leptospirosis in poikilothermic animals has received very little attention, we decided to test reptiles imported into Slovenia. We collaborated with three major Slovenian importers of reptiles who agreed to submit all their dead reptiles for examination. The imported animals were specimens of native species taken from the wild and captive bred species.

The aim of this study was to establish the prevalence of leptospiral antibodies in reptiles, imported into Slovenia with the intention to be pets in close contact with people.

## Methods

A total of 297 reptiles (22 snakes–Serpentes, 210 lizards–Lacertilia and 65 turtles–Testudines) were tested for antibodies against *Leptospira* serovars. Many of the imported animals died during transportation or soon after housing. Most of the carcasses were freshly frozen and periodically sent for post mortem examination. All reptiles whose heart contained blood were tested.

Reptiles were tested for the presence of specific antibodies against pathogenic serovars of *Leptospira interrogans* sensu stricto using the microscopic agglutination test (MAT). The MAT, using live antigens, is the most widely used serological test. It is the reference test according to which all other serological tests are evaluated and used for import/export testing. For optimal sensitivity, it should include representative antigens of all the serogroups known to exist in the region in which the animals are found and, preferably, strains representing all the known serogroups [11]. The test can be used for detecting leptospiral antibodies in various animal species and has been used by several authors investigating the potential role of non-mammalian hosts in the epidemiology of leptospirosis. Evidence that MAT is valid tool for surveying leptospiral infection in terrapins is confirmed by the experimental observation that two different species exhibited homologous seroconversion after infection by the serovar (sv.) Pomona [2].

Reptile hearts containing clotted or unclotted blood were weighed, cut in half and the same weight of buffer was added. Prepared samples were centrifuged for 5 min at 3000 rounds per min and the mixture of buffer and serum was poured off. The dilution of the mixture obtained was estimated as 1:50.

Live cultures of different serovars grown on EMJH medium were used as antigens in the MAT: Grippotyphosa, strain Moskva V, Sejroe, strain Mallerdorf 84, Pomona, strain Pomona, Tarassovi, strain Mitis Johnson, Copenhageni (serological group Icterohaemorrhagiae), strain Wijnberg, Canicola, strain Hond Utrecht IV, Australis, strain Ballico, Bataviae, strain Van Tienen, Saxkoebing, strain MUS 24 and Hardjo, strain Hardjo Bovis. MAT was performed according to standard procedures and the degree of reaction was interpreted by estimating the percentage of agglutinated leptospires. Samples showing titres of ≥ 50 against one or more serovars were considered as positive.

### Risk factors for being seropositive

For the study of risk factors, data on species, region of origin (EU *vs*. non-EU) and feeding were obtained and the chi-squared test was performed using IBM SPSS Statistics program, version 20, with significance level set to *P* < 0.05. If the expected value of one or more cells was less than 5, Fisher’s exact test was performed.

## Results

Details of the tested animals, prevalence and levels of specific leptospiral antibodies are shown in Tables 1, 2, 3.

**Table 1:** **Specific antibodies against pathogen serovars of *Leptospira interrogans *sensu stricto in 12 different snake species**

**Scientific name** **(Common name/Origin)**	**N (tested)**	**N (positive)**	**Serovar**	**Titre rate 1:**
*Platyceps karelini*^1^	4	0	/	/
(Spotted Desert Racer/Pakistan^a^)				
*Platyceps ventromaculatus*^1^	3	1	Tarassovi	100
(Hardwicke’s Rat Snake/Pakistan^a^)				
*Spalerosophis atriceps*^1^	3	0	/	/
(Diadem Snake (Black-headed Royal Snake)/Pakistan^a^)				
*Eryx johnii*^1^	3	3	Grippotyphosa	300
(Brown Sand Boa/Pakistan^a^)			Tarassovi	100
			Grippotyphosa	300
			Tarassovi	100
			Grippotyphosa	200
			Pomona	100
			Copenhageni	50
*Elaphe guttata*^1^	2	0	/	/
(Corn Snake/ EU countries^a^)				
Two aquatic species^1^	2	0	/	/
(undetermined species/Pakistan^a^)				
*Boa constrictor*^1^	1	0	/	/
(Boa Constrictor/EU countries^a^)				
*Vipera ammodytes*^1^	1	1	Copenhageni	400
(Nose-horned Viper/Slovenia^b^)				
*Gongylophis conicus*^1^	1	1	Tarassovi	100
(Rough-tailed Sand Boa/Pakistan^a^)				
*Spalerosophis diadema*^1^	1	0	/	/
(Diadem Snake/Pakistan^a^)				
*Boiga trigonata*^1^	1	0	/	/
(Indian Gamma Snake/Pakistan^a^)				
Total	22	6 (prevalence 27.3%; 95 CI: 8.7–45.9%)		

^a^Snakes were imported from reptile farms.

^b^Originated from Slovenia–native species.

^1^All snakes are carnivores.

**Table 2:** **Specific antibodies against pathogen serovars of *Leptospira interrogans *sensu stricto in 16 different lizard species**

**Scientific name** **(Common name/Origin)**	**N (tested)**	**N (positive)**	**Serovar**	**Titre rate 1:**
*Uromastyx hardwickii*^1^	114	6	Tarassovi	200
(Hardwick’s Spiny-tailed Lizard/Slovenia^a^)			Copenhageni	50
			Grippotyphosa	200
			Grippotyphosa	100
			Grippotyphosa	100
			Grippotyphosa	100
			Grippotyphosa	100
*Eublepharis macularius*^2^	25	10	Tarassovi	200
(Leopard Gecko/Pakistan^b^)			Australis	200
			Grippotyphosa	50
			Tarassovi	200
			Canicola	200
			Grippotyphosa	50
			Tarassovi	200
			Canicola	200
			Grippotyphosa	50
			Tarassovi	200
			Canicola	200
			Grippotyphosa	50
			Tarassovi	200
			Canicola	200
			Grippotyphosa	50
			Grippotyphosa	200
			Grippotyphosa	100
			Grippotyphosa	100
			Grippotyphosa	50
			Grippotyphosa	50
*Gekko gecko*^2^	15	0	/	/
(Tokay Gecko/Pakistan^b^)				
*Uromastyx dispar*^1^	13	8	Grippotyphosa	200
(Sudan Spiny-tailed Lizard/Mali^b^)			Tarassovi	50
			Australis	50
			Grippotyphosa	300
			Grippotyphosa	100
			Grippotyphosa	100
			Grippotyphosa	100
			Grippotyphosa	100
			Grippotyphosa	100
			Grippotyphosa	100
*Iguana iguana*^1^	9	2	Hardjo	1000
(Green Iguana/EU countries^b^)			Tarassovi	300
*Varanus bengalensis*^3^	8	0	/	/
(Bengal Monitor/Slovenia^a^)				
*Varanus niloticus*^3^	6	0	/	/
(Nile Monitor/Slovenia^a^)				
*Uromastyx aegyptia*^1^	5	0	/	/
(Egyptian Spiny-tailed Lizard /Slovenia^a^)				
*Basiliscus plumifrons*^4^	3	3	Grippotyphosa	200
(Green Basilisk/EU countries^b^)			Grippotyphosa	50
			Grippotyphosa	50
*Corucia zebrata*^1^	3	0	/	/
(Solomon Islands Skink/ Solomon Islands^b^)				
*Pogona vitticeps*^1^	3	0	/	/
(Bearded Dragon/EU countries^b^)				
*Varanus flavescens*^3^	2	0	/	/
(Yellow Monitor/Slovenia^a^)				
*Laudakia melanura*^2^	1	1	Grippotyphosa	100
(Black Agama/Pakistan^b^)				
*Chamaeleo calyptratus*^2^	1	0	/	/
(Veiled Chameleon/Slovenia^a^)				
*Teratoscincus scincus*^2^	1	1	Grippotyphosa	50
(Common Wonder Gecko/Pakistan^b^)				
*Varanus indicus*^3^	1	0	/	/
(Mangrove Monitor/ Solomon Islands^b^)				
Total	210		31 (prevalence 14.8%; 95% CI: 10.0–19.6%)	

^a^Originated from Slovenia–species from breeding farms–F1 and F2 generation.

^b^Lizards were imported from reptile farms.

^1^Herbivores, ^2^Insectivores, ^3^Carnivores, ^4^Omnivores.

**Table 3:** **Specific antibodies against pathogen serovars of *Leptospira interrogans *sensu stricto in 8 different turtle species**

**Scientific name** **(Common name/Origin)**	**N (tested)**	**N (positive)**	**Serovar**	**Titre rate 1:**
*Geochelone elegans*^1^	19	0	/	/
(Indian Star Tortoise/Slovenia^a^)				
*Testudo graeca*^1^	18	1	Grippotyphosa	1000
(Spur-thighed Tortoise/Lebanon^b^)			Pomona	300
*Lissemys punctata*^2^	9	0	/	/
(Indian Flap-shelled Turtle/Pakistan^b^)				
*Testudo hermanni*^1^	9	4	Grippotyphosa	400
(Hermann’s Tortoise/Slovenia^a^)			Grippotyphosa	200
			Grippotyphosa	200
			Grippotyphosa	50
*Emys orbicularis*^2^	3	3	Tarassovi	800
(European Pond Turtle/Slovenia^a^)			Copenhageni	50
			Tarassovi	100
			Copenhageni	50
			Tarassovi	1600
			Grippotyphosa	200
			Canicola	200
*Geochelone radiata*^1^	3	0	/	/
(Marginated Tortoise/Slovenia^a^)				
*Testudo horsfieldi*^1^	3	0	/	/
(Horsfield’s Tortoise /Slovenia^a^)				
*Trachemys scripta elegans*^2^	1	1	Tarassovi	200
(Red-eared Slider/Slovenia^a^)				
Total	65	9 (prevalence 13.8%; 95% CI: 5.4–22.3%)		

^a^Originated from Slovenia–species from breeding farms–F1 and F2 generation.

^b^Turtles were imported from reptile farms.

^1^Herbivores, ^2^Omnivores.

Most of the tested snakes were imported in two consignments from Pakistan. Six of 22 tested snakes (27.3%; 95% CI: 8.7–45.9%) were seropositive against four different serovars. Antibodies against sv. Grippotyphosa, Pomona, Tarassovi and Copenhageni were found in three Brown Sand Boas (*Eryx johnii*). Two other snakes (Hardwicke’s Rat Snake (*Platyceps ventromaculatus*) and Rough–tailed Sand Boa (*Gongylophis conicus*)) had antibodies against sv. Tarassovi. Antibodies against sv. Copenhageni were detected in one Nose-horned Viper (*Vipera ammodytes*) originated from Slovenia (Table 1).

Among lizards 31 of 210 tested animals (14.8%; 95% CI: 10.0–19.6%) were seropositive against six different serovars. Antibodies against sv. Hardjo in titre 1:1000 were found in one Green Iguana (*Iguana iguana*). In other lizards, the titres were not higher than 1:300. Among six positive Hardwick’s Spiny-tailed Lizards (*Uromastyx hardwickii*), one was seropositive to sv. Copenhageni and Tarassovi, while antibodies against sv. Grippotyphosa were detected in the others. In an *Uromastyx dispar* imported from Mali, we found antibodies to sv. Grippotyphosa. Antibodies to the same serovar were also found in an *Uromastyx hardwickii* that are offspring of animals imported from Pakistan and in *Basiliscus plumifrons* originating from EU countries.

Among the tested lizards imported from Pakistan, the most seropositive animals were Leopard Geckos (*Eublepharis macularius*). Out of ten seropositive Leopard Geckos, five animals had antibodies against four different serovars (Grippotyphosa, Tarassovi and Canicola and Australis), while antibodies against sv. Grippotyphosa were found in five other Leopard Geckos, one Black Agama (*Laudakia melanura*) and one Common Wonder Gecko (*Teratoscincus scincus*). Among Sudan Spiny-tailed Lizards (*Uromastyx dispar*), imported from Mali, seven seropositive reptiles had antibody titres against sv. Grippotyphosa. Antibodies against sv. Grippotyphosa, Tarassovi and Australis were detected in one lizard. Sv. Grippotyphosa was detected in low titres in three Green Basilisks (*Basiliscus plumifrons*), one Black Agama (*Laudakia melanura*) and one Common Wonder Gecko (*Teratoscincus scincus*). Antibodies against sv. Copenhageni were found in one Hardwicke’s spiny-tailed lizard (*Uromastyx hardwickii*) (Table 2).

Antibodies against five different serovars were detected in nine of 65 terrapins and tortoises (13.8%; 95% CI: 5.4–22.3%). Antibodies against sv. Grippotyphosa and Pomona were found in one Spur-thighed Tortoise (*Testudo graeca*). Hermann’s Tortoises (*Testudo hermanni*) were seropositive (44.4%) to sv. Grippotyphosa. *Testudo hermanni* and *Testudo graeca* imported from Lebanon were housed together and were all positive to the same serovar. Two of three tested European Pond Terrapins (*Emys orbicularis*) from EU countries were seropositive to sv. Tarassovi and Copenhageni, while antibodies against serovars Tarassovi, Grippotyphosa and Canicola were found in one European Pond Terrapin, which had the highest antibody titre (1:1600 against sv. Tarassovi) among all tested turtles. *Emys orbicularis* were kept together with *Trachemys scripta elegans*, also positive to sv. Tarassovi. Among tested turtles 38 animals (6 species) originated from a Slovenian farm and 8 of these were seropositive to serovars Grippotyphosa, Tarassovi, Copenhageni and Canicola (Table 3).

Antibodies against serovars Grippotyphosa, Tarassovi, Copenhageni, Canicola, Sejroe, Hardjo and Australis were found in our investigation. Antibodies against other pathogenic serovars used in the MAT were not detected. Antibody titres higher than 1:800 were established in two *Emys orbicularis*, one *Testudo graeca* and in one *Iguana iguana*.

Table 4 shows potential risk factors for reptiles having leptospiral antibodies. Seroprevalence was statistically similar in snakes irrespectively of origin (*P* = 0.708). All snakes were imported from Pakistan. Among snakes from EU, one originated from Slovenia and 3 were imported from other EU countries.

**Table 4:** **Results of the potential risk factors among seropositive and seronegative snakes, lizards and turtles for having antibodies against *Leptospira *sp**

**Variable**	**N (tested)**	**N (positive)**	**Prevalence (%)**	**CI (95%)**	**P**
**Origin**					
**Snakes**	22	6	27.3	8.7–45.9	0.708*
Other countries: Pakistan	18	5	27.8	7.1–48.4	
EU countries	4	1	25.0	–17.4–76.4	
- originated from Slovenia	1	1	100.0	NR	
- imported from reptile farms	3	0	0.0	NR	
**Lizards**	210	31	14.8	10.0–19.6	<0.001*
Other countries	59	20	33.9	21.8–46.0	
- Pakistan	42	12	28.6	14.9–42.3	
- Mali	13	8	61.5	39.0–84.0	
- Solomon Islands	4	0	0.0	0.0–0.0	
EU countries	151	11	7.3	3.1–11.4	
- originated from Slovenia	136	6	4.4	1.0–7.9	
- imported from reptile farms	15	5	33.3	−6.0–16.0	
**Turtles**	65	9	13.9	5.4–22.3	0.046*
Other countries	27	1	3.7	–3.4–10.8	
- Lebanon	18	1	5.5	–5.0–16.0	
- Pakistan	9	0	0.0	0.0–0.0	
EU countries: originated from Slovenia	38	8	21.1	8.1–34.0	
**Feeding**					
**Lizards**					<0.001
Herbivores	147	16	10.9	5.9–15.9	
Insectivores	43	12	27.9	14.5–41.3	
Carnivores	17	0	0.0	0.0–0.0	
Omnivores	3	3	100.0	NR	
**Turtles**					0.070
Herbivores	52	5	9.6	1.6–17.6	
Omnivores	13	4	30.8	5.7–55.9	

*: indicate statistical difference (P) between EU countries and other countries.

*NR,* not relevant (used for the groups with N < 4).

Only 7.3% (95% CI: 3.1–11.4%) of lizards from EU were positive against leptospiral antibodies (1.4% of lizards originated from Slovenia), whereas the seroprevalence in lizards from other countries was significantly higher (33.9%; 95% CI: 21.8–46.0%) (*P* < 0.001). Positive animals were imported from Pakistan and Mali, whereas none of the animal from Solomon Island was positive. On the other hand, a higher seroprevalence was observed in turtles from EU (all originated from Slovenia) than from turtles from other countries (Pakistan and Lebanon) (*P* = 0.046).

Feeding was found as a risk factor for seropositivity in lizards (*P* < 0.001). None of the carnivores but all of omnivores were seropositive.

## Discussion

Our study shows presence of antibodies against *Leptospira* sp. in snakes, lizards and turtles imported into Slovenia. Results on snakes are comparable with the findings of other studies [3,6,7,12-15] where 24.4% to 41% of snakes were found seropositive to one or more leptospiral serovars, whereas we found a seroprevalence of 27.3%. We found specific antibodies for the serovars Grippotyphosa, Tarassovi, Copenhageni and Pomona (Table 1), whereas only antibodies against sv. Grippotyphosa has been reported previously [3].

Published serological data on leptospirosis in lizards are very rare. Pleško *et al.*[4] found that 29.7% of 37 lizards belonging to only two species (European Green Lizard (*Lacerta viridis*) and the sand lizard (*Lacerta agilis*) were seropositive to sv. Sejroe, Canicola, Grippotyphosa and Bataviae. We examined 210 lizards belonging to 16 different species and found 14.8% seropositive animals with titres ranging from 1:50 to 1:1000. We found antibodies to six different serovars (Grippotyphosa, Tarassovi, Canicola, Australis, Copenhageni and Hardjo) (Table 2). Comparing to the results of Pleško *et al*. [4], antibodies to four additional serovars were found (Tarassovi, Australis, Copenhageni and Hardjo), whereas serovars Sejroe and Bataviae were not found in our study.

To our knowledge, there are only a few studies concerning turtles. In our study the seroprevalence in turtles was 13.8% and antibodies against the five sv. Grippotyphosa, Tarassovi, Canicola, Pomona and Copenhageni at titre levels between 1:50 and 1:1600 were found (Table 3). In *Trachemys scripta elegans* we found antibodies against sv Tarassovi which is comparable with the results of Glosser *et al*. [16] who found serologic evidence of sv. Tarassovi in Red-eared Sliders (*Trachemys scripta elegans*) (prevalence 91.3%; 95% CI: 83.2–99.4%) trapped in sewage settling ponds. Silva *et al*. [17] investigated the presence of antileptospiral agglutinins in 40 captured freshwater terrapins belonging to two species and 11 animals were found positive (prevalence 27.5%; 95% CI: 15.0–40.0%). The most common predicted infecting serovar was Bataviae. In another species of freshwater turtle (European Pond Turtle–*Emys orbicularis*) antibodies against serovars Tarassovi, Copenhageni, Grippotyphosa and Canicola were found. Except for sv. Tarassovi [16,17], antibodies against these serovars does not seem to have been reported in freshwater turtles.

Origin of reptiles was identified as a risk factor for presence of leptospiral antibodies.

Although the prevalence of leptospiral antibodies did not differ significantly between snakes from EU and other countries (all imported snakes were from Pakistan), the results should be interpreted with caution due to the large confidence interval that originated from the low number of snakes from EU (n = 4).

Low prevalence with leptospiral antibodies was found in lizards originated from Slovenia, whereas one third of imported lizards from other reptile farms in EU were seropositive (Table 4). Origin from Pakistan and Mali were found as a risk factor for leptospiral antibodies in comparison with origin within EU countries.

The situation is different in turtles. A significant higher prevalence of antibodies was found in turtles from Slovenia (all turtles examined originated from Slovenia) in comparison to other countries. Slovenian turtle breeders observed a large number of Brown rats and house mice around their farms. These rodents are frequently carriers and lifetime spreaders of sv. Grippotyphosa in Slovenia and there is an obvious risk of indirect transmission of the infection to tortoises on the premises as previously reported [10]. All other tortoises strictly separated from *Testudo hermanni* and *Testudo graeca* with no possibility of contact with rodents, were negative.

Turtles were imported from Pakistan and Lebanon, but only one animal from Lebanon was positive. We suggest turtles originating from Pakistan and Lebanon are at low risk for having leptospiral antibodies.

It is difficult to interpret the zoonotic risk based on the serological response found in our study as this may just reflect a previous infection. Paired serum samples were not obtainable due to the study design being founded on dead reptiles and culturing or molecular detection methods were not applied. However, other studies have shown that the relationship between *Leptospira* and the herpetofauna of biotope may be an essential part of the epidemiology of leptospirosis [6,7,15]. Successful experimental infection of reptiles with detected leptospiremia and contact infection of pen mates [2] and successful cultural evidence [16] indicate that the role of these animals in maintaining leptospirosis in the environment and the possibility of spreading the infection should be taken more seriously.

## Conclusions

We found antibodies against seven pathogenic serovars of *L. interrogans* sensu stricto in 46 of 297 tested reptiles. Origin from outside the EU represents a risk factor for being seropositive for leptospirosis, especially in lizards. These results indicate that reptiles may be considered as potential reservoirs of leptospires. Further studies to investigate the possible carrier status and evaluate the possible risk of transmission of pathogenic leptospires from reptiles to other animals and humans are needed.

## Competing interests

The authors declare that they have no competing interests.

## Authors’ contributions

AD, AVR and KV have been involved in the initial design of the study and protocols. RLK has been responsible for the serological work. AD has been the main responsible for data analysis. PZ has been involved in interpretation of the results and was responsible for statistical evaluation. All authors have contributed substantially to the editing of the manuscript. All authors read and approved the final version of the manuscript.
